# Genetics of diaphragmatic hernia

**DOI:** 10.1038/s41431-021-00972-0

**Published:** 2021-10-08

**Authors:** Yannick Schreiner, Thomas Schaible, Neysan Rafat

**Affiliations:** Department of Neonatology, University Children’s Hospital Mannheim, University of Heidelberg, Mannheim, Germany

**Keywords:** Respiratory tract diseases, Mutation, Genetics research

## Abstract

Congenital diaphragmatic hernia (CDH) is a life-threatening malformation characterised by failure of diaphragmatic development with lung hypoplasia and persistent pulmonary hypertension of the newborn (PPHN). The incidence is 1:2000 corresponding to 8% of all major congenital malformations. Morbidity and mortality in affected newborns are very high and at present, there is no precise prenatal or early postnatal prognostication parameter to predict clinical outcome in CDH patients. Most cases occur sporadically, however, genetic causes have long been discussed to explain a proportion of cases. These range from aneuploidy to complex chromosomal aberrations and specific mutations often causing a complex phenotype exhibiting multiple malformations along with CDH. This review summarises the genetic variations which have been observed in syndromic and isolated cases of congenital diaphragmatic hernia.

## Introduction

Congenital diaphragmatic hernia (CDH), accounting for roughly 8% of all major congenital malformations is a severe physical deformity associated with high morbidity and mortality and occurs in less than 5 cases in 10,000 births [1, 2]. Due to improved treatment options survival has greatly improved up to 88% [3] but long-term morbidity commonly remains an important issue [4]. CDH is caused by a discontinuity in the diaphragm allowing abdominal organs to penetrate into the thoracic cavity, to interfere with heart and lung development thus causing a series of severe pathophysiologic events: pulmonary hypoplasia, pulmonary hypertension (PH) following increased pulmonary vascular resistance (PVR) and cardiac impairment are hallmarks of CDH [5]. Notably, most cases occur sporadically being referred to as isolated or nonsyndromic, respectively. However, genetic causes ranging from aneuploidy to complex chromosomal aberrations and specific mutations have long been discussed to explain a proportion of cases of CDH often along with additional malformations [6, 7]. This review summarises the recurrent genetic variations which have been observed in syndromic and isolated cases of congenital diaphragmatic hernia, including copy number variations, point mutations and the role of vitamin A homoeostasis and signalling pathways.

## Normal diaphragm and lung development

During embryonic development, the intraembryonic coelom arises as a precursor of the body’s cavities. The cranially located right and left limb of the coelum, referred to as pleuropericardial canals, adhere to the growing lungs as they are pushed away by the growing respiratory organs. Thereby, thin folds appear on the right and left side of the heart: the pleuropericardial folds cranially and the pleuroperitoneal folds caudally. Together with the transverse septum, the esophageal mesentery, and the posthepatic mesenchymal plate (PHMP), the latter orchestrate the formation of the diaphragm [8, 9]. If the pleuroperitoneal folds close insufficiently or if a weakness within the musculature or connective tissue remains, the congenital diaphragmatic hernia can occur. For a detailed explanation on lung and diaphragm development, please refer to the supplemental material.

## Genetic variations in congenital diaphragmatic hernia

### The significance of copy number variations (CNVs)

Several studies have examined the association between the CNVs and certain diseases and provide evidence for both causative and predisposing relations [10–12]. To what extent these genomic imbalances could potentially contribute to CDH development has long been unclear but recent scientific evidence sheds light on this relatively new genetic approach, revealing a subset of as much as 10% of CDH cases as attributable to CNVs [13]. Notably, most CNVs that have so far been identified to play a role in the development of CDH are deletions, which in the context of haploinsufficiency are considered deleterious in the process of diaphragm development. However, several CDH-related chromosomal loci are affected by copy number gains. To date, CNVs putatively responsible for CDH could be found in numerous loci. Zhu et al. compared 196 CDH cases to 987 healthy controls and identified six CNVs significantly associated with CDH (Suppl. Table 1) [14]. Of these, a gain in copy number of parts of *HLX1* found in five patients with CDH is easiest to associate to the developing diaphragm since Hlx could be determined in the murine embryonic septum transversum and diaphragm [15, 16]. Also, Hlx-signal was colocalized with Myod in myoblasts of the upper somites [15] suggesting its contribution to myogenesis. These findings make *HLX1* an interesting candidate gene in CDH.

### Disruption of essential signalling pathways can lead to CDH

Disp1 is a protein similar to the hedgehog-receptor Ptch acting in the Sonic Hedgehog (Shh) signalling pathway [17]. In mouse studies, Disp1 is specifically expressed in the PPFs of the developing premature diaphragm [18]. Yet, Shh-expressing cells were shown to neither contribute to the muscular, nor to the nonmuscular part of the developing murine diaphragm in fate-mapping studies, and Ptch1 was not found in the PPFs [18]. Therefore, the Disp1 function remains unknown in diaphragm development. However, murine Shh-signalling contributes to phrenic nerve outgrowth [18], which in neural tube cells is executed by the transcription factors Gli2 and Gli3 [19] and a promotor element of *NR2F2* (*COUP-TFII*) responsive to Shh-signalling was identified in a murine cell line [20]. A similar activation of *NR2F2* during human diaphragm development has yet to be uncovered. But since Shh-signalling is crucial for embryonic lung [21] and phrenic nerve development and mutations of *GLI2* and *GLI3* are described in human cases of CDH [22, 23], Shh-signalling could still influence the developing human diaphragm. Furthermore, Kantarci et al. discovered two distinct deletions on chromosome 1 encompassing both *HLX* and *DISP1* associated with CDH that consistently remain of uncertain significance [18]. In contrast, there is evidence for functional Wnt/β-catenin-signalling in the developing diaphragm upstream of which acts WT1 to promote β-catenin expression and cell proliferation especially in the posterior diaphragm [24]. In the embryo, canonical Wnt-signalling is an essential driver for development and requires multiple cellular proteins like WNT5A and FZD2 [25]. Consistently, there is evidence suggesting disrupted Wnt-signalling could contribute to CDH in a male infant harbouring a deletion in 17q12.2 encompassing *FZD2* [26]. In line with the importance of Wnt/β-catenin-signalling, Scott et al. reported on a male infant exhibiting a deletion on chromosome 11p13 covering *WT1* and *PAX6* [27]. Notably, *PAX6* is required in myogenesis and its contribution to the CDH phenotype could not be excluded.

### Low copy repeats at 8p23 are responsible for CNVs leading to CDH

A mutational hotspot for structural chromosomal aberrations (e.g., translocations, inversions) and microaberrations (e.g., point mutations, base deletions, indels) is the short arm of chromosome 8. Yu et al. and Keitges et al. determined CNVs at 8p23.1 in CDH patients with additional ventricular septal defect (VSD), atrial septal defect (ASD) or incomplete atrioventricular canal defect, or both ASD and VSD [28, 29]. In one case, only two genes, namely *GATA4* and *NEIL2*, were affected by the deletion pinpointing on another CDH candidate region located at 8p23. *NEIL2* is involved in base excision repair and is therefore not considered a good CDH candidate. At that time, loss of function mutations of *GATA4* were already associated to cardiac anomalies [30, 31] and today we know that *GATA4* is important for both embryonic cardiac and lung development [32, 33]. Furthermore, the examination of diaphragm development in mice in which *Gata4* was specifically deleted in the PPFs identified muscle connective tissue fibroblasts as a source of CDH in *Gata4*-deficient organisms [34]. Developing CDH can therefore be associated with *GATA4* deficiency in the nonmuscular mesenchyme. Additionally, Wat at al. report on a male infant with a heterozygous de novo del(8p23.1) mutation encompassing *GATA4*, exhibiting multiple cardiac anomalies along with anterior, right-sided CDH [35] similar to those observed in *Gata4*^+/∆ex2^ mice [33]. Notably, his monozygotic twin brother presented with cardiac defects only, omitting CDH despite the presence of a similar mutation also involving *GATA4* [35]. These findings from animal studies suggest a striking value of *GATA4* transcriptional activity in the developing diaphragm. Notably, both deletions encompassed *SOX7*, another transcription factor that acts upstream of *GATA4* and might multiply the disrupting effects of *GATA4* deficiency either if *SOX7* itself is deleted or affected by missense mutations [36].

### NR2F2 (COUP-TFII) – Key element in CDH?

*NR2F2* (*COUP-TFII*), mentioned previously as a potential target of Shh-signalling in neural cells, was also found to be expressed in the murine nonmuscular mesenchyme of the PPFs and is frequently affected by deletions in patients with CDH [37–39]. Consistently, *Nr2f2*-knockout mice develop diaphragmatic hernia [40]. Interestingly, multiple pathways unite upon this essential transcription factor (Supplemental Figure 1), which was found to colocalize with WT1, likewise expressed in PPF connective tissue [37] and to interact with GATA4 and FOG2 [41]. We are unable to provide possible mechanisms of action for all of the CNVs described in impaired diaphragm development. However, a comprehensive list of additional CNVs associated with CDH is presented in Supplemetary Table 1.

## Point mutations associated with CDH SNVs

### The pinpoint for candidate genes encompassed by CNVs

Besides complex CNVs, also single nucleotide variations (SNVs) are associated with cases of CDH. Some of these mutation sites correspond to regions where CNVs have already been detected thus highlighting significant genes within the deleted or duplicated loci, e.g., *NR2F2* [42] or *GATA4* [43] (Suppl. Table 2). Similar to GATA4, GATA6 is another zinc-finger transcription factor involved particularly in heart and lung organogenesis [44, 45] and interestingly, CDH cases exhibiting *GATA6* variants were also reported [46, 47]. Notably, GATA6 expression is restricted to the mesenchymal part of the PPFs [48]. The activity levels of GATA4 and GATA6 are both modulated by the zinc-finger transcription factor FOG2 (also known as ZFPM2) thus activating or repressing transcriptional activity. Hence, mutations affecting *FOG2* could also interfere with the diaphragm, heart, and lung development. At E13.5, FOG2 and GATA4 were found to be expressed in nuclei in cells of the PPF. Conversely at E16.5, GATA4 and FOG2 were also determined in the muscularized diaphragm though only FOG2 switched localisation to cytosolic [37]. To our knowledge, Ackerman et al. were the first to discover *FOG2* variants in association with CDH [49]. In their experiments, *Fog2*^*+/-*^ mice displayed pulmonary dysplasia and the absence of an accessory right lobe along with an intact but muscular posterior and peripheral diaphragm [49]. Furthermore, *Fog2*^*+/-*^ mice exhibited severely downregulated HGF expression in the PPFs. How FOG2 might influence the developing diaphragm is not yet fully understood. However, the authors concluded, that impaired migration of muscle cell progenitors due to defective HGF/c-MET signalling led to the non-muscularized diaphragm and hypothesised HGF expression might be directly regulated by FOG2. Also, FOG2 is thought to act as a corepressor of NR2F2 [50]. Consistently, SNVs in *MET* were found in cases of CDH [23, 51] supporting the concept of defective migration as a cause for high diaphragmatic vulnerability.

## The role of vitamin A homoeostasis and signalling pathways

There is scientific consensus about the importance of vitamin A and its derivatives in embryonic lung development [52, 53]. Tissue-specific retinaldehyde dehydrogenases (RALDHs) generate retinoic acids (RAs) in two different isoforms, namely all-trans retinoic acid and 9-cis retinoic acid both binding to different intracellular receptors (RAR or RXR), which then form heterodimers and translocate to the nucleus, where they act as transcription factors by binding to specific retinoic acid response elements (RAREs, RXREs). During alveologenesis, RALDH2 (ALDH1A2) and ALDH1 specifically expressed in lung tissue are of significant importance for generating RAs [54], which are thought to perpetuate mesodermal proliferation, to induce FGF10 and to affect shh-signalling during the period of initial budding and branching morphogenesis [52, 55, 56]. Also, retinoic acids act upstream of homeobox transcription factors like HOXB4 to promote lung differentiation [57]. Interestingly, there are mutated variants of *HOXB4* associated with CDH [23]. Supplemental Figure 1 provides an overview on vitamin A’s metabolism and target genes in lung development. The observation, that vitamin A deficiency during rat dams’ pregnancy did not only impede lung development but did also cause diaphragm defects in the pups led to the hypothesis that vitamin A signalling would be involved in its morphogenesis [58, 59]. Indeed, Aldh1a2, retinol-binding protein Rbp, and Rarβ were found to be strongly expressed in the PPFs of rat pups [60–62] and generated *Rar*-knockout strains exhibiting diaphragmatic defects additional to pulmonary hypoplasia further highlight the importance of vitamin A in diaphragm development [61]. Conversely, Clugston et al. evaluated the presence of Rarα, Rarγ and Rxrα but did not determine Rarβ transcripts in the developing murine diaphragm [63]. Subsequently performed studies on human newborns revealed a reduction of retinol and RBP in the chord blood by up to 50% in CDH cases [64]. In consistence with the emerging concept of vitamin A-dependent diaphragm development, it was shown NR2F2 (COUP-TFII) transcription factors could act as repressors of and in turn be regulated by retinoic acid signalling [65, 66] and by this, a possible mechanism in CDH pathogenesis was revealed. Supporting the concept of essential vitamin A signalling in diaphragm development, both point mutations in and CNVs of genes involved in vitamin A metabolism and signalling were described affecting several levels of retinoid signalling. Starting at the top of the signalling cascade, SNVs in *STRA6* (encoding a membrane receptor involved in the uptake of vitamin A) and a CNV covering *STRA6* potentially compromising vitamin A uptake in affected cells were discovered [67, 68]. Furthermore, *ALDH1A2* was found to be affected by duplications on chromosome 9 in CDH cases [69, 70]. Interestingly, one of those duplications also covered the retinoid acid receptors *RARA* and *RXRA*, the terminal endpoints of retinoid signalling [69]. Given the numerous mutations in members of the retinoid signalling pathway discovered in CDH patients (Supplementary Table 1 and 2), we provide evidence for its significant contributions to diaphragm development and CDH pathogenesis.

## Discussion

We comprehensively summarise point mutations and copy number variations described in the literature associated with CDH. Both have contributed equally to the identification of CDH candidate genes and are nowadays indispensable in its deep diagnosis. However, declaring detected genes explicitly causative would be a bold statement with regards to the complexity of genetics and organogenesis during embryonic development. Because deleted or duplicated chromosomal loci often carry many different genes, a disease-causing genotype-phenotype association is a difficult venture. Also, the deletion or duplication of promotor regions or regulatory units could possibly escape our notice. Moreover, the fact that in some cases unaffected relatives harbour the same genetic anomaly further underlines the complexity of CDH pathogenesis. Nevertheless, our summary of genetic findings associated with CDH offers the possibility for clinicians and geneticist to classify rare mutations in affected infants as potentially relevant, and subsequently to screen parents for the respective genetic variant and offer genetic counselling. By doing so, future pregnancies rated high-risk upon the presence of certain mutations could be closely monitored in order to improve the outcome of the newborn. The development of diaphragmatic hernias can be considered the endpoint of a single (rare), dual, or even multiple hit event in multiple tissues or the interaction between cells of different ancestry. We consider mutational events in the pathogenesis of CDH as being of predisposing character as it remains difficult to make single specific events solely responsible for the development of diaphragmatic defects. Mutated genes can be assigned to multiple signalling pathways (e.g., retinoid, hedgehog, wnt), play a role in various areas of cellular homoeostasis or are crucial during embryonic development. The majority resembles transcription factors of which some had been exhaustively investigated and their presence in the premature murine diaphragm or pleuroperitoneal folds have been determined. However, we think more transcription factors and proteins could be found in the premature diaphragm and further extend the spectrum of CDH candidate genes and possible pathogenic backgrounds. There seems to be more independent factors explaining why some individuals with certain genetic anomalies do develop CDH and others do not. Likewise, genetics alone could hardly explain the local restriction of CDH defects. Furthermore, there is evidence for essential vitamin A signalling in the developing primitive lungs and diaphragm. Disruption of the retinoid signalling pathway would therefore be able to constitute a basis for an in vivo applicable dual hit hypothesis, suggesting that lung hypoplasia could in part occur independently of a diaphragmatic defect or even precede it. In the literature, possible pathways by which vitamin A could impede diaphragm development are poorly discussed. Hence, its contribution to the pathogenicity of CDH remains largely unknown. By demonstrating RA-inducible NR2F2 activity, at least one possible mechanism of RA-regulated CDH development has been revealed. Further research needs to be done to verify if there are interactions between retinoic acid and other transcription factors known to be crucial for diaphragm development.

## Supplementary information

Revised Supplements_clean version

## Data Availability

All data generated or analysed during this study are included in this published article [and its supplementary information files].
