# Supplementary material for: Genetics of diaphragmatic hernia
Source: Eur J Hum Genet. 2021 Oct 8;29(12):1729–33. doi: 10.1038/s41431-021-00972-0 (PMC8632982; doi:10.1038/s41431-021-00972-0)
Supplement: Supplementary file 1 — Revised Supplements_clean version [file 41431_2021_972_MOESM1_ESM.docx]

**Supplements**

| **Supplementary Table 1**. Copy number variations (CNVs) described in individuals with congenital diaphragmatic hernia (CDH). Each line resembles one patient. In some cases, a maximal and minimal deleted region from the analysis of one patient is given. Mutations in different genes all affecting the same individual are indicated by exponents. Each exponent resembles one patient. ^∆^ Although not explicitly stated by the authors, the listed genes map to the deleted region and are presumably associated with CDH (Callaway et al.). ** Variants of unknown significance (if the extent of contribution to the pathogenicity of CDH was doubted or critically discussed by the authors). n/a = not available.^4^ karyoytpe: 46,XX(1) / 47,XX,+mar(1), ^5^ karyotype: 46,XY,der(12)t(11;12)(q23;q24). | | | | | | |
| --- | --- | --- | --- | --- | --- | --- |
| **Banding** | **Deletion / Duplication Range** | **Size** | **Gain/Loss** | **Inheritance** | **Candidate Genes / Possible effects** | **Reference** |
| 1q12-qter | 142,693,887-247,249,716 | 104,555,829 | Gain | de novo | HLX, MYOG, LMNA, CACNA1S, AGT, PBX1, CRABP2, DISP1 | (1) |
| 1q41 | 221,052,740-221,054,346 | 1,606 | Gain | n/a | HLX, HLX-AS1 (non-coding RNA) | (2) |
| 1q41-q42.12 | 218,227,107-224,441,046 | 6,213,93 | Loss | de novo | DISP1, HLX | (3) |
|  | 223,076,895-225,311,293 (minimal)  223,073,839-225,318,623 (maximal) | 2,234,398  2,244,784 | Loss | de novo | DISP1, WDR26, TP53BP2 | (4) |
|  | 218,297,696 – 225,150,422 | 6,852,726 | Loss | n/a | DISP1, HLX | (3) |
| 1q44 | 249,126,046-249,238,916 | 112,870 | Loss | n/a | ZNF672, ZNF692, PGBD2 | (2) |
| 1p13.3-p12 | 107,379,866-119,388,162 | 12,008,296 | Loss | de novo | KCNA2 | (1) |
| 1p21-p12 | 103,114,239-118,470,102 | 15,355,863 | Loss | de novo | n/a | (5) |
| 1p22.2 | 92,027,785-92,129,888** | 102,103 | Gain | paternal | TGFBR3 | (6) |
| 2q35-q36.1 | 221,191,674-223,302,616 | 2,110,942 | Loss | paternal | PAX3 | (7) |
| 2p16.3 | 50,890,607-51,079,734 | 189,127 | Loss | paternal | NRXN1 | (8) |
| 3p26.3-p25.2 | 66,478-12,304,371 | 12,237,893 | Loss | de novo | n/a | (2) |
| 4q21.23 | 86,595,913-87,070,050 | 474,137 | Loss | maternal | ARHGAP24 | (9) |
| 4q28.3-qter | n/a | n/a | Gain | maternal | n/a | (10) |
| 4q35.2-qter | 185,963,614-189,037,586 | 3,073,972 | Gain | paternal | FAT1, regulation of downstream targets | (11) |
| 4p15.2-p14 | 22,819,570-36,306,519 | 13,486,949 | Gain | de novo | RBPJ, PPARGC1A | (6) |
| 4p16.3 | 11,942-143,314 | 131,372 | Loss | inherited | ZNF595, ZNF718 | (2) |
|  | 85,743-18,953,893 (minimal)  1-18,984,868 (maximal) | 18,868,150  18,984,865 | Loss | de novo | FGFR3, FGFRL1, CTBP-AS2, NSD2, ZNF141, MAEA, CPLX1, CTBP1 | (12) |
|  | 1-9,945,373 | 9,945,372 | Loss | n/a | FGFRL1, NSD2 | (13) |
|  | 1-2,336,628 | 2,336,627 | Loss | de novo | FGFR3, FGFRL1, CTBP-AS2, NSD2, ZNF141, MAEA, CPLX1, CTBP1^∆^ | (14) |
| 5q31.3-q35.5 | 142,548,354-180,696,806^7^ | 38,148,452 | Gain | maternal | NDST1, ADAM19, NSD1, MAML1 | (15) |
| 5p15.2 | 12,674,767-12,754,177 | 79,410 | Loss | n/a | LINC01194 (non-coding RNA) | (2) |
| 6q27 | 169,866,304-169,884,311 | 18,007 | Loss | n/a | TCTE3 | (16) |
| 6p25.3-p25.2 | 1,190,693-2,762,165 | 1,571,562 | Loss | de novo | FOXC1, FOXF2 | (1) |
| 7p22.3 | 52,899-2,004,194^1^ | 1,951,295 | Loss | paternal | PDGFA | (1) |
| 8q22.3-q23.1 | 105,914,640-106,907,764 (minimal)  105,880,441-106,922,626 (maximal) | 993,124  1,042,185 | Loss | paternal | FOG2 | (4) |
| 8q22.3-q24.23 | 104,516,737-136,746,871 (minimal)  104,508,082-136,809,150 (maximal) | 32,230,134  32,301,068 | Loss | de novo | FOG2, OXR1, EXT1, TRPS1 | (4) |
| 8q23.1 | 106,417,969-106,704,253 | 286,284 | Loss | paternal | FOG2 | (17) |
|  | 106,812,277-107,420,029 (minimal)  106,800,200-107,511,467 (maximal) | 607,752  711,267 | Loss | maternal | FOG2, OXR1 | (4) |
| 8p22-p23.3 | 958,312-15,160,490 | 14,202,178 | Loss | n/a | GATA4 | (18) |
| 8p23.1 | 8,043,620-11,883,409 | 3,839,789 | Loss | de novo | GATA4, SOX7 | (1) |
|  | 8,850,913-11,796,333 (minimal)  6,436,314-12,788,647 (maximal) | 2,945,420  6,352,333 | Loss  Loss | de novo  de novo | GATA4 | (19) |
|  | 11,530,791-11,657,980 | 127,189 | Loss | maternal | GATA4, NEIL2 | (20) |
|  | 11,583,841-11,898,980 | 315,139 | Loss | n/a | GATA4, NEIL2, FDFT1, CTSB, DEFB136, DEFB135, DEFB134 | (20) |
| 8p23.2-p22 | 192,262-15,227,167 | 15,034,905 | Loss | de novo | GATA4, SOX7 | (1) |
| Tetrasomy 9p | n/a | n/a | Gain | de novo | ALDH1A2, RARA, RXRA | (21) |
| 9p22.3 | 14,892,957-14,941,672 (minimal)  14,869,861-14,955,988 (maximal) | 48,715  86,127 | Loss | maternal | FREM1 | (22) |
| 10q26 | 114,660,279-114,838,014 | 177,735 | Loss | maternal | TCF7L2 | (9) |
| 10q26.3-qter | 132,932,808-135,434,178^7^ | 2,501,370 | Loss | maternal | n/a | (15) |
| 11q13.2-q23.2 | 65,440,233-114,273,551 | 48,833,318 | Gain | de novo | n/a | (2) |
| 11q23.1-q25 | 116,187,461-134,452,384^2^ | 18,264,924 | Gain | maternal | HYLS1 | (1) |
| 11q23.3-qter | n/a^5^ | ~19 Mb | Gain | maternal | ROBO3, ROBO4, CDON | (23) |
| 11p12-p15.1 | n/a | n/a | Loss | n/a | PAX6, WT1 | (24) |
| 11p15 | 2,349,955-20,939,091 | 18,589,136 | Gain | de novo | affects ICR2 (imprinting control region 2),  KCNQ1, CDKN1C | (25) |
| 12p13.33-p11.22 | 20,691-28,622,103^1^ | 28,601,412 | Gain | paternal | FGF6 | (1) |
| 13q12.12 | 22,439,339-23,808,774** | 1,369,435 | Gain | paternal | n/a | (6) |
| 13q14.3-q33.3 | 51,620,432-108,613,709 | 56,993,277 | Gain | de novo | n/a | (6) |
| 13q33.3-q34 | 107,348,518-114,142,980 | 6,794,462 | Loss | de novo | COL4A1, COL4A2  COL4A2 | (1) |
| 15q11.2 | 20,305,686-20,691,555 | 385,869 | Loss | not maternal | n/a | (7) |
| 15q13.1-q13.2 | 29,213,743-30,300,265 (minimum)**^6^  28,525,505-30,349,558 (maximum)**^6^ | 1,086,522  1,824,053 | Gain | paternal | APBA2, FAM189A1, NSMCE3, TJP1 (additional findings not considered causative for CDH) | (12) |
| 15q21.3 | 57,529,846-58,949,448 | 1,419,602 | Gain | maternal | ALDH1A2 | (26) |
| 15q24.1-q24.2 | n/a appx. 70.3-74.6 Mb | ~4.3 Mb | Loss | de novo | STRA6, MPI, MAN2C1, LMAN1L, CYP11A1, CYP1A1 | (27) |
|  | 70,397,546-74,208096 | 3,810,550 | Loss | de novo | n/a | (28) |
| 15q25.2 | 80,999,115-82,497,270 | 1,498,156 | Loss | de novo | HOMER2, BNC1 | (1) |
|  | 80,422,638-82,856,949^3^ | 2,434,311 | Loss | de novo | BNC1, BTBD1 | (6) |
|  | 83,214,012-84,812,634 (minimal)  82,664,440-84,928,631 (maximal)  83,214,012-85,721,698 (minimal)  82,557,992-85,815,602 (maximal) | 1,598,622  2,264,191  2,507,686  3,257,610 | Loss  Loss | de novo  not maternal | BTBD1, HDGFRP3, BNC1 | (29) |
|  | 81,011,052-82,602,711 | 1,591,659 | Loss | de novo | BNC1, BTBD1 | (6) |
| 15q26.1-q26.2 | n/a | ~5 Mb | Loss | n/a | NR2F2, CHD2, RGMA, SIAT8B | (30) |
| 15q26.1-qter | n/a | n/a | Loss | not maternal | MEF2A, IGF1R | (31) |
| 15q26.2 | 94,221,024-95,920,254^4^ | 1,699,230 | Loss | de novo | NR2F2, SPATA8 | (6) |
| 15q26.2-q26.3 | 95,715,936- 100,168,859  89,236,682-100,168,718  85,342,977-100,338,915 | 4,452,923  10,932,036  14.995.938 | Loss  Loss  Loss | n/a | IGF1R, ARRDC4 | (32) |
| 16p11.2 | 32,403,182-34,759,850 | 2,356,668 | Gain | n/a | TP53TG3E, TP53TG3B, TP53TG3F, TP53TG3C | (2) |
|  | 28,833,437-29,046,252 | 212,815 | Loss | de novo | ATXN2L, TUFM, SH2B1, ATP2A1, RABEP2, CD19, NFATC2IP, SPNS1, LAT, hsa-MiR-4517 | (33) |
|  | 29,559,861-30,102,955 | 543,094 | Loss | de novo | TBX6 | (6) |
|  | 29,652,999-30,199,351 (minimal)  29,350,831-30,332,522 (maximal)  29,502,653-30,274,073 | 546,352  981,691  771,420 | Loss  Loss | de novo  de novo | TBX6 | (4) |
| 17q12 | 34,813,719-36,278,623  31,890,483-33,281,801 | 1,464,904  1,391,318 | Loss  Loss | n/a  n/a | AATF, ACACA, DDX52, DUSP14, GGNBP2, HNF1B, LHX1, MYO19, DHRS11, MRM1, C17orf78, PIGW, SYNRG, TADA2A, ZNHIT3 | (2, 34) |
|  | 34,437,475-36,243,XXX | ~1.8 Mb | Loss | de novo | PIGW | (35) |
|  | 31,897,638-33,362,422  32,058,213-33,668,463 | 1,464,784  1,610,250 | Loss  Loss | de novo  de novo | LHX1 | (1) |
| 17q12.2 | 42,633,066-42,650,463 (minimal)  42,622,151-42,657,504 (maximal) | 17,397  35,353 | Loss | de novo | FZD2 | (4) |
| 17p12-p11.2 | 15,742,070-20,501,924 | 4,759,854 | Loss | not maternal | RAI1 | (36) |
| 17p12 | 14,039,023-15,382,903**^3^ | 1,343,880 | Loss | de novo | n/a | (6) |
| 17p13.3 | 1,656-714,933** | 713,277 | Gain | maternal | n/a | (6) |
| 18q22.1 | 62,306,291-64,627,510 | 2,321,219 | Loss | maternal | CDH19, DSEL, TXNDC10 | (37) |
| 18p11.32 | 138,763-76,111,164 | 75,972,401 | Gain | de novo | n/a | (1) |
| 22q11.1-q11.21 | 14,432,516-18,709,056^2^ | 4,276,541 | Gain | maternal | BID, TBX1 | (1) |
| 22q11.21 | 18,138,802-18,460,940 | 322,138 | Loss | maternal | TBX1 | (7) |
| Xq12-q13.1 | 67,435,556- 68,458,832 (male karyotype)  67,331,076-68,633,303 | 1,023,276  1,302,227 | Gain  Gain | de novo | EFNB1 | (38) |
| Xq13.1 | 67,899,816-68,240,036 | 340,220 | Gain | n/a | EFNB1 | (18) |
| Xq26.2 | 132,688,097-132,808,036 (male karyotype) | 119,939 | Loss | maternal | GPC3 | (39) |

| **Supplementary Table 2.** Point mutations described in individuals with congenital diaphragmatic hernia. Locus according to Ensemble data bank. Each line resembles one patient. Two or more Mutations affecting the same gene in one individual are connected by ‘AND’. Mutations in different genes all affecting the same individual are indicated with exponents. Each exponent resembles one patient. Mutations affecting the same gene within one individual are connected by ‘AND’. If X-chromosomes are affected, karyotypes are presented in parenthesis to account for hemizygousity in male infants. ** Variants of unknown significance (if the extent of contribution to the pathogenicity of CDH cannot be predicted or if their predicted impacts were contradictory. n/a = not available. ^7^ accompanied by COL6A2 microdeletion of ~ 92 kb of unknown significance. ^8^ accompanied by dup16p13.11. ^9^ accompanied by del8p23. | | | | | |
| --- | --- | --- | --- | --- | --- |
| **Locus** | **Gene** | **Mutation (karyotype)** | **Effect** | **Inheritance** | **Reference** |
| 1q21.3 | POGZ | c.2849dupC | p.V951S*fs* | n/a | (40) |
| 1q23.3 | PBX1 | c.700C>T | p.R234W | n/a | (41) |
| 1q25.1 | RC3H1 | c.3136-2delA | splice site mutation considered highly disruptive | n/a | (42) |
| 1q41 | HLX | c.950A>C (homozygous) | p.D317A | maternal **AND** paternal | (43) |
|  |  | c.704C>T**  c.35C>T  c.53C>T  c.517G>T | p.A235V  p.S12F  p.S18L  p.D173Y | not maternal  n/a  n/a  n/a | (44) |
|  |  | c.27C>G** | p.F9L | maternal | (3) |
|  |  | c.1172C>A** | p.T391K | n/a | (42) |
|  | DISP1 | c.4412C>G** | p.A1471G | de novo | (3) |
|  |  | c.3287T>C**  c.3395G>A** | p.M1096T  p.R1132Q | paternal  n/a | (42) |
| 1q44 | ZNF692 | c.974+1G>T | splice site mutation | n/a | (2) |
|  | PGBD2 | c.1447T>C | p.Y483H | n/a | (2) |
| 1p31.1 | PRKACB | c.277G>T | p.R93X | de novo | (45) |
| 1p33 | SLC5A9 | c.172G>T | p.R58C | de novo | (45) |
| 1p36.13 | PAX7 | c.1451G>A  c.335C>T  c.638G>A **AND** c.644G>A | p.R484H  p.P112L  p.R215H **AND** p.R213H | maternal  n/a  paternal | (42) |
| 1p36.33 | B3GALT6 | c.929A>G **AND** c.795A>C | p.Y310C **AND** p.E265D | n/a | (40) |
| 2q14.2 | INHBB | c.C1055G | p.T352R | de novo | (45) |
|  | GLI2 | c.4471C>A**  c.4628G>A  c.2159G>A  c.293T>A  c.1294G>A  c.1975G>A** | p.P1491T  p.R1543H  p.R720H  p.I98N  p.V432M  p.G659R | maternal  not maternal  maternal  n/a  not maternal  n/a | (42) |
| 2q31.1 | LRP2 | c.6978dupG  c.4855C>A | p.T2327D*fs*X4  p.L1619I | maternal  n/a | (46)  (42) |
|  |  | c.3667+1G>A **AND** c.5390A>G | n/a **AND** p.N1797S | n/a | (40) |
| 2q35 | DES | c.638C>T^1^ | p.A213V | paternal | (47) |
|  | STK36 | c.3641C>G **AND** c.3578C>G | p.S1193* **AND** p.S1214* | n/a | (42) |
| 2q36.1 | PAX3 | c.944C>A^1^  c.764C>T **AND** c.767C>T** | p.T315K  p.A256V **AND** p.A255V | paternal  not maternal | (47)  (42) |
| 2p22.1 | SOS1 | c.3298G>T | p.D1100Y | n/a | (40) |
| 2p23.3 | DNMT3A | c.1867dupT^7^ | p.Y623L*fs**7 | not maternal | (48) |
| 3p12.3 | ROBO1 | c.4352G>T **AND** c.4517G>T **AND** c.4652G>T** | p.R1451I **AND** p.R1551I **AND** p.R1506I | paternal | (42) |
|  | ROBO2 | c.1793T>C **AND** c.1841T>C  c.551G>A **AND** c.599G>A**  c.3904C>T **AND** c.3856C>T | p.I598T **AND** p.I614T  p.R200H **AND** p.R184H  p.R1286W **AND** p.R1302W | paternal  n/a  n/a | (42) |
| 3p13 | FOXP1 | c.1718_1722+8del | n/a | de novo | (40) |
| 3p14.3 | WNT5A | c.561delC (homozygous) | p.Y188M*fs**31 | maternal **AND** paternal | (49) |
| 3p21.31 | SMARCC1 | c.3170delC | p.P1057*fs**5 | n/a | (42) |
| 3p24.2 | RARB | c.1159C>T  c.1159 C>A  c.355C>T **AND** c.1201_1202insCT  (compound heterozygous) | p.R387C  p.R387S  p.R119* **AND** p.I403S*fs**15 (compound heterozygous) | de novo  de novo  paternal **AND** maternal | (50) |
| 4q12 | PDGFRA | c.2889C>G  c.2410C>T | p.L967V  p.R804* | n/a  n/a | (51)  (42) |
| 4q21.3 | PTPN13 | c.571G>T | p.E191* | n/a | (42) |
| 4q21.21 | FRAS1 | c.2389G>A^2^  c.9806G>A^3^**  c.6323A>T^4^ | p.E797K  p.R3269Q  p.D2108V | maternal  maternal  paternal | (52) |
| 4p15.2 | ADGRA3 | c.754C>T | p.Q252* | n/a | (42) |
| 4p16.3 | FGFRL1 | c.442C>T**  c.1474G>A** | p.R148C  p.V492M | paternal  paternal | (42)  (12) |
|  | NSD2 | c.904C>T**  c.2807T>C** | p.P302S  p.V936A | maternal  paternal | (12) |
|  | ZNF141 | c.487C>T | p.R163C | n/a | (12) |
|  | FGFR3 | c.985G>A | p.V329I | maternal | (12) |
| 5q14.3 | RASA1 | c.1103-1G>T | n/a | paternal | (40) |
| 5q22.3 | CDO1 | c.259delG | p.D87*fs* | de novo | (45) |
| 5q35.1 | SLIT3 | c.2333C>T  c.2552A>G | p.T778M  p.H851R | paternal  paternal | (42) |
|  |  | c.1525G>T (homozygous) | p.R510C | both parents heterozygous | (53) |
| 5p13.1 | FYB | c.972dupG **AND** c.1002dupG | p.P335*fs**43 **AND** p.P325*fs**43 | n/a | (42) |
| 5p13.2 | NIPBL | c.5524C>T | nonsense mutation | n/a | (54) |
| 5p15.1 | MYO10 | c.2722G>T^5^ | p.E908X | de novo | (55) |
| 6p21.31 | SCUBE3 | c.1988_1991delGAGA | p.R663*fs**37 | n/a | (42) |
| 6p25.3 | FOXF2 | c.1274G>A  c.628G>A** | p.G425E  p.V210M | paternal  n/a | (42) |
|  | FOXC1 | c.1474_1479delGCGGCG | p.A488_A489del | n/a | (42) |
| 7q11.23 | PTPN12 | c.77G>T | p.T26M | de novo | (45) |
| 7q31.2 | MET | c.2975C>T^1^  c.3029C>T **AND** c.2975C>T  c.3202_3203insATGT **AND** c.3256_3257insATGT  c.948A>G | p.T992I  p.T1010I **AND** p.T992I  p.V1088*fs* **AND** p.V1070*fs*  p.I316M | paternal  n/a  paternal  maternal | (47)  (42) |
| 7p14.1 | GLI3 | c.2726C>G^2^  c.223C>A  c.200G>T  c.2179G>A** | p.A909G  p.P75T  p.G67V  p.G727R | maternal  n/a  not maternal  maternal | (52)  (42) |
| 7p21.1 | TWIST | c.445C>T | p.L149F | paternal | (56) |
| 8q12.2 | CHD7 | c.1018A>G**  c.4811G>C**  c.3299G>A  c.4775G>A  c.2840G>A  c.7469C>T** | p.M340V  p.S1604T  p.R1100H  p.R1592Q  p.R947Q  p.S2490L | maternal  paternal  maternal  paternal  maternal  paternal | (42) |
| 8q13.3 | EYA1 | c.65C>T **AND** c.164C>T  c.1321G>A **AND** c.1327G>A **AND** c.1426G>A | p.T22M **AND** p.T55M  p.D441N **AND** p.D443N **AND** p.D476N | de novo  paternal | (42) |
| 8q21.13 | ZFHX4 | c.7307C>G  c.9002G>T  c.5327A>G | p.S2436W  p.G3001V  p.E1776G | not paternal  maternal  maternal | (42) |
| 8q22.3 | FOG2 | c.1396_1399dup  c.89A>G  c.292G>A  c.679A>G  c.960C>G  c.2501A>G  c.2593A>G  c.3086A>T  c.1632G>A**  c.172G>T**  c.3186delC** | p.Y467*fs**23  p-E30G  p.D98N  p.I227V  p.H320Q  p.K834R  p.K865E  p.K1029I  p.M544I  p.E58*  p.N1062*fs**23 | maternal  de novo  n/a  paternal  paternal  n/a  n/a  n/a  maternal  paternal  n/a | (17) |
|  |  | n/a | p.R112X | de novo | (57) |
|  |  | c.2107A>C  c.2527A>G | p.M703L  p.T843A | n/a  n/a | (51) |
|  |  | c.663_664dup  c.89A>G | p.R222H*fs**49  p.E30G | maternal  paternal | (41) |
|  |  | c.757_761dup | p.C255*fs* | n/a | (40) |
| 8p23.1 | GATA4 | c.-458+5G>A** | mutation of splice donor site of non-coding exon 1 | n/a | (58) |
|  |  | c.1103C>T | p.P368L | de novo | (41) |
|  |  | c.754C>T  c.848G>A | p.R252W  p.R283H | paternal  de novo | (59)  (41) |
|  |  | c.1037C>T**  c.1129A>G** | p.A346V  p.S377G | paternal  n/a | (60) |
|  | SOX7 | c.506C>G**  c.698G>A**  c.799G>A** | p.T169S  p.R233H  p.G267S | paternal  paternal  not maternal | (60) |
|  |  | n/a**  n/a**  n/a | p.A350S  p.G267S  p.C253Y | paternal  not maternal  maternal | (61) |
|  | NEIL2 | c.161C>G **AND** c.344C>G  c.-46+1678delA **AND** c.22delA  c.144-3320T>C **AND** c.241T>C **AND** c.424T>C** | p.S54C **AND** p.S115C  p.R8*fs**51 **AND** p.R8*fs**40  p.W142R **AND** p.W81R | not maternal  maternal  paternal | (42) |
|  |  | n/a** | p.S115C | not maternal | (61) |
|  | MCPH1 | c.586delC (homozygous) | p.Q196*fs* | n/a | (40) |
| 9q33.3 | GAPVD1 | c.2809C>T** | p.R937W | not de novo | (50) |
|  | PBX3 | c.182C>T **AND** c.407C>T  c.182C>T **AND** c.407C>T  c.965T>C **AND** c.1190T>C** | p.A61V **AND** p.A136V  p.A61V **AND** p.A136V  p.L322P **AND** p.L397P | maternal  paternal  maternal | (42) |
| 9q34.12 | ABL1 | c.1066G>A | p.A356T | de novo | (40) |
|  |  | c.734A>G | p.Y245C | de novo | (40) |
|  |  | c.352T>C | p.W118R | de novo | (40) |
| 9p13.3 | TLN1 | c.G98A | p.R33H | de novo | (45) |
| 9p13.13 | DNASE2 | c.473T>C | p.L158P | n/a | (42) |
| 9p22.3 | FREM1 | c.5334+1G>A | mutation of splice donor site of intron 28 | paternal | (22) |
|  |  | c.1394G>C^1^ | p.G465A | paternal | (47) |
| 9p24.1 | PLPP6 | c.824T>A | p.V275E | de novo | (45) |
| 10q11.23 | CHAT | c.84G>C  c.53_54delAG  c.8C>T  c.400C>T **AND** c.508C>T **AND** c.754C>T**  c.605T>G **AND** c.251T>G **AND** c.359T>G | p.R28S  p.E20*fs*  p.P3L  p.H134Y **AND** p.H252Y **AND** p.H170Y  p.M120R **AND** p.M202R **AND** p.M84R | paternal  not maternal  paternal  maternal  not maternal | (42) |
| 10q26.13 | CTBP2 | c.590G>C  c.811G>A**  c.524_525delAG  c.1147G>A **AND** c.2767G>A** | p.R197T  p.E271K  p.Q175*fs*  p.A383T **AND** p.A923T | not paternal  n/a  maternal  n/a | (42) |
|  | FGFR2 | n/a | p.S252W | n/a | (62) |
| 10p11.22 | ZEB1 | c.2722+1G>A **AND** c.2782+1G>A **AND** c.2785+1G>A **AND** c.2734+1G>A **AND** c.2731+1G>A **AND** c.2581+1G>A | splice site mutations considered highly disruptive | n/a | (42) |
| 11q12.2 | MYRF | c.2518C>T^6^ | p.R840X | de novo | (63) |
|  |  | c.1328A>C | p.Q443P | de novo | (64) |
|  |  | c.3239dupA | p.E1081Gf*s* | de novo | (40) |
|  |  | c.350_366delinsT | p.G117V*fs* | not maternal | (40) |
| 11q13.1 | FBLN4 | c.169G>A (homozygous) | P.E57K | maternal **AND** paternal | (65) |
| 11q13.4 | DHCR7 | c.964-1G>C**^6^ | n/a | paternal | (63) |
| 11q24.2 | ROBO4 | c.569G>C^4^ | p.G190A | maternal | (52) |
| 11p13 | WT1 | c.1097G>A | p.R366H | n/a | (66) |
|  |  | c.1316G>A | p.R439H | de novo | (67) |
| 11p15.1 | MYOD1 | c.122G>T  c.355C>T | p.R41L  p.R119W | n/a  paternal | (42) |
| 11p15.4 | CTR9 | c.3085G>A^9^ | p.A1029T | de novo | (67) |
| 12q13.12 | KMT2D | c.9931C>T **AND** c.10101G>T | p.Q3311X **AND** p.L3367F | de novo | (68) |
|  |  | c.10258dupA | p.I3420*fs* | de novo | (40) |
|  |  | c.13543dupA | p.R4515*fs* | de novo | (40) |
|  |  | c.7613dupT | p.Q2540S*fs* | de novo | (40) |
|  |  | c.1967delT | p.L656*fs* | n/a | (40) |
| 12q13.2 | SMARCC2 | c.1651-2A>G | n/a | not maternal | (40) |
| 12q24.13 | PTPN11 | c.175A>G | p.T59A | de novo | (67) |
| 12q24.21 | TBX5 | c.1115C>T  c.331G>T **AND** c.181G>T  c.331G>T **AND** c.181G>T  c.316A>G **AND** c.166A>G** | p.S372L  p.D111Y **AND** p.D61Y  p.D111Y **AND** p.D61Y  p.I56V **AND** p.I106V | n/a  n/a  paternal  n/a | (41)  (42) |
| 13q13.1 | BRCA2 | c.4965C>G **AND** c.7007G>C | p.Y1655Ter **AND** p.R2336P | n/a | (40) |
| 13q13.3 | FREM2 | c.4031G>A **AND** c.4558G>T  c.5938_5940delCTT (homozygous)  c.4031G>A^2^  c.4031G>A^3^  c.4994C>T^4^ | p.R1344H **AND** p.R1520W  p.L1980del  p.R1344H  p.R1344H  p.S1665F | maternal **AND** paternal  maternal **AND** paternal  paternal  paternal  maternal | (52) |
| 14q11.2 | MMP14 | c.829C>T  c.958G>A | p.R277W  p.D320N | paternal  maternal | (42) |
| 14q22.2 | BMP4 | c.592C>T | p.R198X | de novo | (69) |
| 14q23.1 | SIX4 | c.1927A>G** | p.M643V | maternal | (42) |
| 14q24.3 | DLST | c.297_298del | p.99_100del | de novo | (45) |
| 14q32.11-32.12 | CCDC88C | c.3807_3809delinsACCT **AND** c.3967-?_4112-? | p.G1270P*fs**53 p.L1323R*fs**10 | maternal paternal | (70) |
| 15q21.1 | FBN1 | c.4969_4970insA^1^ | p.I1657N*fs**30 | paternal | (47) |
|  |  | c.4786C>T | p.R1596Ter | de novo | (40) |
| 15q21.3 | NEDD4 | c.529A>G | p.S177G | maternal | (42) |
|  | TCF12 | c.1808G>A | p.R603Q | de novo | (40) |
| 15q24.1 | STRA6 | c.145_147delC (homozygous)  c.1931C>T (homozygous)  c.269C>T **AND** c.961A>C (homozygous) | p.G50A*fs*X22  p.T644M  p.P90L **AND** p.T321P | maternal **AND** paternal  inherited  inherited | (71) |
| 15q24.2 | SIN3A | c.1570_1577del | p.Y524V*fs**26 | de novo | (45) |
| 15q26.1 | RGMA | c.196G>A**  c.362C>G** | p.A66T  p.T121S | n/a | (72) |
|  | CHD2 | c.5128C>T** | p.R1710W | n/a | (72) |
|  | SIAT8B | c.674A>G** | p.K225R | n/a | (72) |
|  | KIF7 | c.2432T>G**  c.1486G>C** | p.V811G  p.V496L | paternal  not maternal | (42) |
|  | FANCI | c.2422A>T | p.K808Ter | maternal | (40) |
| 15q26.2 | NR2F2 (COUP-TFII) | c.-60C>T  c.1096C>T  c.92_98delGCCCGCC | mutation in 5’ UTR, binding site for E2F1 transcription factor  p.R213C  p.P33A*fs*Ter77 | n/a  n/a  de novo | (73) |
|  |  | c.442+1G>A |  | n/a | (41) |
|  |  | n/a | p.L264S*fs*Ter55 | de novo | (74) |
|  |  | c.71C>A^8^ | p.S24X | de novo | (67) |
|  | ARRDC4 | c.1171C>T** | p.R391W | paternal | (72) |
|  |  | c.417G>C**  c.649G>A** | p.Q139H  p.E217K | paternal  n/a | (42) |
| 15q26.3 | IGF1R | c.3061G>A  c.3601G>A  c.1297G>T | p.V1021M  p.V1201I  p.D433Y | n/a  n/a  maternal | (42) |
| 16q24.1 | FOXC2 | c.563_573del | p.P188*fs* | de novo | (40) |
| 16q24.3 | ANKRD11 | c.1372C>T | p.R458Ter | paternal | (40) |
| 16p11.2 | TBX6 | c.484G>A  c.815G>A | p.G162S  p.R272Q | de novo  n/a | (42) |
| 17q12 | AATF | c.1085A>G | p.Y362C | n/a | (2) |
|  | DDX52 | c.1061C>T  c.1042C>G  c.247_249dupAGG  c.14A>G | p.A354V  p.R348G  p.R83dup  p.D5G | n/a  n/a  n/a  n/a | (2) |
|  | GGNBP2 | c.1609_1611delAGG | p.K538del | n/a | (2) |
|  | MYO19 | c.2146G>A  c.1905+1G>A  c.446A>G  c.425C>T | p.A916T  splice site mutation  p.Y149C  p.S142F | n/a  n/a  n/a  n/a | (2) |
|  | DHRS11 | c.205T>A | p.C69S | n/a | (2) |
|  | PIGW | c.705C>G  c.883C>T | p.H235Q  p.R295W | n/a  n/a | (2) |
|  | SYNRG | c.2835T>G  c.590G>A | p.F1024L  p.G197D | n/a  n/a | (2) |
|  | TADA2A | c.143G>A | p.R48Q | n/a | (2) |
| 17q21.2 | RARA | c.353C>T **AND** c.368C>T | p.T123M **AND** p.T118M | paternal | (42) |
| 17q21.32 | HOXB4 | c.548A>G  c.727C>T | p.Y183C  p.R243W | n/a  n/a | (42) |
| 17p11.2 | RAI1 | c.2461G>A (hemizygous) | p.P664P (conservative) | n/a | (36) |
| 17p12 | ELAC2 | c.1444G>T **AND** c.1441G>T **AND** c.1324G>T | p.E442* **AND** p.E482* **AND** p.E481* | n/a | (42) |
| 17p13.1 | TP53 | c.375+1G>A^9^ | splice site | de novo | (67) |
| 18q11.2 | GATA6 | c.1516+4A>G | weakens the strength of the 5’ splice donor site in intron 5 | de novo | (75) |
|  |  | c.1366C>T  c.712G>T  c.1071delG | p.R456C  p.G238*  p.V358C*fs*34* | de novo  maternal  de novo | (76)  (45) |
|  |  | c.1291C>T | p.Q431* | maternal | (77) |
| 18q21.33 | PIGN | c.1574+1G>A (homozygous) | splice site mutation, dysfunctional protein | maternal **AND** paternal | (78) |
|  |  | c.421dup (homozygous) | p.I141N*fs**10 | Maternal **AND** paternal | (79) |
|  |  | c.1966C>T **AND** c.1674+1G>C | p.E656X **AND** splice site mutation | n/a | (80) |
| 18p11.31 | TGIF1 | c.707A>T **AND** c.260A>T **AND** c.320A>T **AND** c.362A>T**  c.90G>A | p.Q87L **AND** p.Q107L **AND** p.Q121L **AND** p.Q236L  p.W30* (isoform a) | paternal  n/a | (42) |
| 19q13.2 | DLL3 | c.1091A>G | p.N364S | maternal | (42) |
|  | ILF1 | c.1966_1967insC **AND** c.1954_1955insC  c.2192C>A **AND** c.2180C>A** | p.G656*fs**37 **AND** p.G652*fs*  p.P727H **AND** p.P731H | not maternal  maternal | (42) |
|  | RPS19 | c.380G>A | p.G127E | n/a | (81) |
|  | LTBP4 | c.3554delA (homozygous)  c.791delC **AND** c.2570_2571delGCinsAA  c.820T>G (homozygous)  c.2570_2571delGCinsAA **AND** c.4128insC | p.Q1185*fs*X1211  p.P264*fs*X300 **AND** p.C857X  p.C274G  p.C857X **AND** p.P1376*fs*X1403 | maternal **AND** paternal  maternal **AND** paternal  n/a  not maternal | (82) |
| 19p13.2 | SMARCA4 | c.2936G>A | p.R979Q | de novo | (40) |
| 19p13.3 | ADAT3 | c.587C>T **AND**  c.586_587delinsTT | p.A196V  p.A196L | maternal  paternal | (40, 83) |
|  | LONP1 | c.1325G>T | p.T442M | de novo | (45) |
| 20q13.12 | SLC2A10 | c.67G>A (homozygous) | p.G23S | n/a | (40) |
| 20q13.13 | ARFGEF2 | c.3326G>A | p.R1109H | de novo | (45) |
| 20q13.33 | GATA5 | c.424T>C | p.Y142H | n/a | (41) |
|  | COL20A1 | c.3153+2T>C  c.2581_2582delGG | splice site mutation considered highly disruptive  p.G861*fs**28 | n/a  n/a | (42) |
| 21q22.12 | RUNX1 | c.11A>G** | p.D4G | maternal | (42) |
| 22q11.21 | TBX1 | c.928G>A | p.G310S | maternal | (41) |
| 22q13.2 | EP300 | c.3592T>C | p.Y1198H | n/a | (40) |
|  |  | c.2660C>T (Mosaic 12%) | p.T887I (Mosaic) | de novo | (40) |
| 22q13.33 | ALG12 | c.165C>A **AND** c.437G>A | p.Y55Ter **AND** p.R146Q | n/a | (40) |
|  |  | c.437G>A **AND** c.930_931delAC | p.R146Q **AND** p.R311*fs* | n/a | (40) |
| Xq13.1 | EFNB1 | c.712delG (female karyotype) | p.V238W*fs*X21 | paternal | (84) |
|  |  | c.432delG (hemizygous; male karyotype)  c.151_153delGTG (hemizygous; male karyotype) | p.L145W*fs*X14  p.V51del | maternal  maternal | (85) |
|  | MED12 | c.5691_5692delGT (female karyotype) | p.Y1898*fs* | de novo | (40) |
| Xq25 | STAG2 | c.1840G>T (female karyotype) | p.R614X | de novo | (45) |
| Xq26.2 | GPC3 | c.1573+1G>A (hemizygous; male karyotype)  c.187C>T (hemizygous; male karyotype) | mutation in intro 7 predicted to be damaging  p.Q63* | maternal  maternal | (39) |
| Xp11.4 | BCOR | c.626C>T (hemizygous; male karyotype)** ^5^ | p.S209L | maternal | (55) |
|  | DDX3X | c.1304T>C (sex unknown) | p.L435P | de novo | (40) |
| Xp11.23 | PORCN | c.1077C>G (heterozygous; female karyotype) | p.Y359X | not maternal | (86) |
|  | HDAC6 | c.40C>T (male karyotype) | p.R14X | de novo | (67) |
| Xp21.2 | NR0B1 | c.1142T>C** (hemizygous; male karyotype) | p.L381P | maternal | (87) |
| Xp22.12 | PDHA1 | c.1035_1036dupGA (female karyotype) | p.I346R*fs* | not maternal | (40) |
| Xp22.2 | CLCN4 | c.43G>A (female karyotype) | p.D15N | de novo | (45) |
|  | HCCS | c.308_309insAGT (sex unknown) | p.V103dup | de novo | (40) |


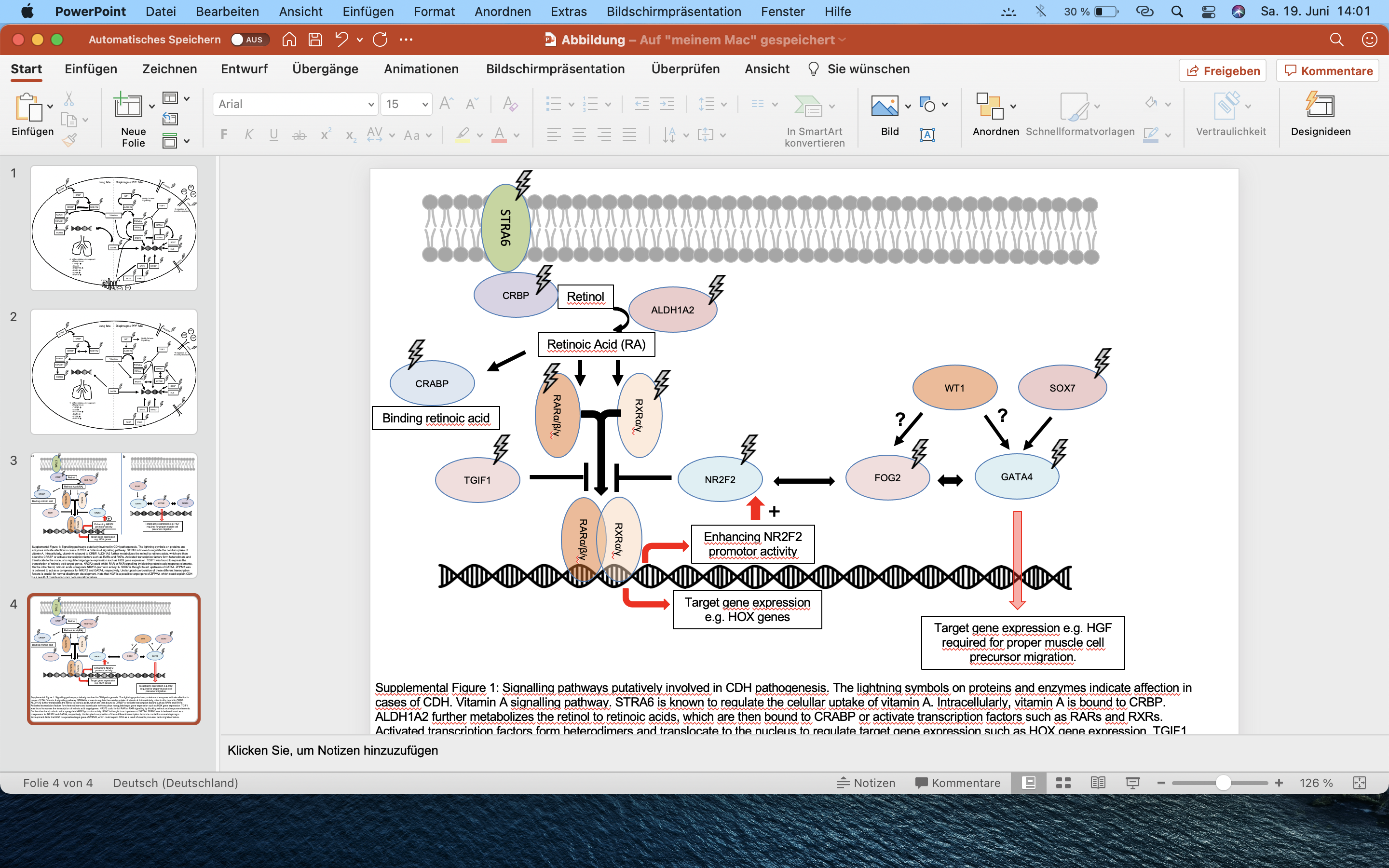


**Supplemental Figure 1:** Signalling pathways putatively involved in CDH pathogenesis. The lightning symbols on proteins and enzymes indicate affection in cases of CDH. Left half: Vitamin A signalling pathway. STRA6 is known to regulate the cellular uptake of vitamin A (88). Intracellularly, vitamin A is bound to CRBP. RALDH1 (also known as ALDH1A2) further metabolizes retinol to retinoic acids (89), which are then bound to CRABP or activate transcription factors such as RARs and RXRs. Activated transcription factors form heterodimers and translocate to the nucleus to regulate target gene expression such as HOX gene expression (90). TGIF1 was found to repress the transcription of retinoic acid target genes (91). NR2F2 (also known as COUP-TFII) could inhibit RAR or RXR signalling by blocking retinoic acid response elements (92). In turn, NR2F2 promotor activity can be upregulated by retinoic acids (93). Right half: SOX7 was shown to regulate GATA4 expression (60). FOG2 is believed to act as a corepressor for NR2F2 (94). WT1 was found to colocalize with FOG2, GATA4 and NR2F2 in the PPFs of the premature diaphragm (95) though its contribution to CDH pathogenicity remains uncertain. Note that HGF is a possible target gene of FOG2 (57), which could explain CDH resulting from muscle precursor cells failing to migrate to the diaphragm. Undisrupted cooperation of these different transcription factors seems to be crucial for normal diaphragm development.

# Normal diaphragm and lung development

During the third week of development, the embryo starts to fold both in a cranio-caudal and transversal direction thus forming the intra-embryonic horseshoe-shaped body cavity known as the intra-embryonic coelom. This continuous tube with its apex located around the heart will give rise to the pericardial, pleural and peritoneal cavity. At the beginning of the fourth week, the lungs will start to develop as a ventral diverticulum from the cranial primitive endodermal foregut. As it begins to grow downwards into the future pleural cavity, separated from the oesophagus only by a thin oesophagotracheal septum, it will form the rudiments of the two principal and five lobar bronchi. Later on, the trachea will be separated from the oesophagus altogether except for its top where the connection between them remains to form the laryngeal cavity. The cranially located right and left limb of the intra-embryonic coelom underneath the pericardial cavity, structures referred to as the pleuropericardial canals adhere to the growing lungs as the visceral pleura as they are pushed away by the growing respiratory organs. Thereby, two thin folds appear on the right and left side of the heart: the pleuropericardial folds. They segregate the heart from the lungs and later serve as guiding scaffoldings for the phrenic nerve to reach the diaphragm. At the same time, the growing lungs cause another fold to appear at its caudal end in separation to the peritoneal cavity: the pleuroperitoneal folds (PPF). Those structures at the right and left bottom of the lungs divide the pleural cavity from the peritoneal cavity. Furthermore, the cranio-caudal folding of the embryo relocates the cranially formed primitive heart into its chest cavity. As embryonal development proceeds, mesenchymal tissue arises underneath the heart, and dorsal to the liver, structures referred to as the septum transversum and the posthepatic mesenchymal plate (PHMP), respectively, the former eventually giving raise to the central tendon of the diaphragm (96). For the intact diaphragm to evolve, PPFs, PHMP, oesophageal mesentery and septum transversum need to fuse. Notably for muscularization of the premature diaphragm, muscle precursor cells originating from the cervical somites C3-C5 begin to migrate to the PPFs at E9.5 thus preceding phrenic nerve outgrowth which is first detectable at E11.25 and also originates from nerve segments C3-C5 (97). Notably, the tyrosine kinase receptor c-Met (MET) is expressed by diaphragm muscle precursor cells, which together with its ligand hepatocyte growth factor (HGF) expressed along their migratory path seems to play a crucial role in the processes of long distance migration and differentiation (98). Evidence is provided by c-Met^-/-^ mice failing, which accurately develop dermomyotome, but nevertheless fail to develop a muscularized diaphragm due to impaired migration (98, 99), thus emphasizing the importance of intact HGF/c-Met signalling.

However, if PPFs close insufficiently or if fusion of any of the components is prohibited, a weakness within the musculature or connective tissue remains and congenital diaphragmatic hernia can occur. Due to its organogenesis of different components leading to distinct fusion sites, the diaphragm exhibits certain predilection sites of high vulnerability being predominantly affected by herniation. The most frequent Bochdalek hernia (100) can either be right- or left-sided and occurs between the costal and lumbar parts of the muscle though left-sided Bochdalek hernia is more common (101, 102). A protrusion of abdominal content between the sternum and the right costal part of the diaphragm is called a Morgagni hernia or Larrey hernia if it occurs on the left side, respectively.

# References (for supplementary material)

1. Yu L, Wynn J, Ma L, Guha S, Mychaliska GB, Crombleholme TM, et al. De novo copy number variants are associated with congenital diaphragmatic hernia. Journal of medical genetics. 2012;49(10):650-9.

2. Zhu Q, High FA, Zhang C, Cerveira E, Russell MK, Longoni M, et al. Systematic analysis of copy number variation associated with congenital diaphragmatic hernia. Proceedings of the National Academy of Sciences. 2018;115(20):5247-52.

3. Kantarci S, Ackerman KG, Russell MK, Longoni M, Sougnez C, Noonan KM, et al. Characterization of the chromosome 1q41q42. 12 region, and the candidate gene DISP1, in patients with CDH. American Journal of Medical Genetics Part A. 2010;152(10):2493-504.

4. Wat MJ, Veenma D, Hogue J, Holder AM, Yu Z, Wat JJ, et al. Genomic alterations that contribute to the development of isolated and non-isolated congenital diaphragmatic hernia. Journal of medical genetics. 2011;48(5):299-307.

5. Ibrahim M, Hunter M, Gugasyan L, Chan Y, Malhotra A, Sehgal A, et al. Interstitial deletion of chromosome 1 (1p21. 1p12) in an infant with congenital diaphragmatic hernia, hydrops fetalis, and interrupted aortic arch. Clinical case reports. 2017;5(2):164.

6. Brady P, DeKoninck P, Fryns J-P, Devriendt K, Deprest J, Vermeesch J. Identification of dosage‐sensitive genes in fetuses referred with severe isolated congenital diaphragmatic hernia. Prenatal diagnosis. 2013;33(13):1283-92.

7. Stark Z, Behrsin J, Burgess T, Ritchie A, Yeung A, Tan TY, et al. SNP microarray abnormalities in a cohort of 28 infants with congenital diaphragmatic hernia. American Journal of Medical Genetics Part A. 2015;167(10):2319-26.

8. Bermudez-Wagner K, Jeng LJ, Slavotinek AM, Sanford EF. 2p16. 3 microdeletion with partial deletion of the neurexin-1 gene in a female with developmental delays, short stature, and a congenital diaphragmatic hernia. Clinical dysmorphology. 2013;22(1):22-4.

9. Veenma D, Brosens E, De Jong E, Van De Ven C, Meeussen C, Cohen-Overbeek T, et al. Copy number detection in discordant monozygotic twins of Congenital Diaphragmatic Hernia (CDH) and Esophageal Atresia (EA) cohorts. European Journal of Human Genetics. 2012;20(3):298.

10. Celle L, Lee L, Rintoul N, Savani RC, Long W, Mennuti MT, et al. Duplication of chromosome region 4q28. 3‐qter in monozygotic twins with discordant phenotypes. American journal of medical genetics. 2000;94(2):125-40.

11. Bogs T, Kipfmüller F, Kohlschmidt N, Gembruch U, Müller A, Reutter H. Familial tetrasomy 4q35. 2 associated with congenital diaphragmatic hernia and unilateral renal agenesis: a case report. Journal of medical case reports. 2016;10(1):76.

12. Callaway DA, Campbell IM, Stover SR, Hernandez-Garcia A, Jhangiani SN, Punetha J, et al. Prioritization of Candidate Genes for Congenital Diaphragmatic Hernia in a Critical Region on Chromosome 4p16 using a Machine-Learning Algorithm. Journal of pediatric genetics. 2018;7(04):164-73.

13. Tautz J, Veenma D, Eussen B, Joosen L, Poddighe P, Tibboel D, et al. Congenital diaphragmatic hernia and a complex heart defect in association with Wolf-Hirschhorn syndrome. Am J Med Genet A. 2010;152(11):2891-4.

14. Casaccia G, Mobili L, Braguglia A, Santoro F, Bagolan P. Distal 4p microdeletion in a case of Wolf‐Hirschhorn syndrome with congenital diaphragmatic hernia. Birth Defects Research Part A: Clinical and Molecular Teratology. 2006;76(3):210-3.

15. Chen C-P, Huang J-P, Chen S-W, Chern S-R, Wu P-S, Wu F-T, et al. Prenatal diagnosis of concomitant distal 5q duplication and terminal 10q deletion in a fetus with intrauterine growth restriction, congenital diaphragmatic hernia and congenital heart defects. Taiwanese Journal of Obstetrics and Gynecology. 2020;59(1):135-9.

16. Teshiba R, Masumoto K, Esumi G, Nagata K, Kinoshita Y, Tajiri T, et al. Identification of TCTE3 as a gene responsible for congenital diaphragmatic hernia using a high-resolution single-nucleotide polymorphism array. Pediatric surgery international. 2011;27(2):193-8.

17. Longoni M, Russell M, High F, Darvishi K, Maalouf F, Kashani A, et al. Prevalence and penetrance of ZFPM2 mutations and deletions causing congenital diaphragmatic hernia. Clinical genetics. 2015;87(4):362-7.

18. Srisupundit K, Brady PD, Devriendt K, Fryns JP, Cruz‐Martinez R, Gratacos E, et al. Targeted array comparative genomic hybridisation (array CGH) identifies genomic imbalances associated with isolated congenital diaphragmatic hernia (CDH). Prenatal diagnosis. 2010;30(12‐13):1198-206.

19. Wat MJ, Shchelochkov OA, Holder AM, Breman AM, Dagli A, Bacino C, et al. Chromosome 8p23. 1 deletions as a cause of complex congenital heart defects and diaphragmatic hernia. American Journal of Medical Genetics Part A. 2009;149(8):1661-77.

20. Keitges EA, Pasion R, Burnside RD, Mason C, Gonzalez‐Ruiz A, Dunn T, et al. Prenatal diagnosis of two fetuses with deletions of 8p23. 1, critical region for congenital diaphragmatic hernia and heart defects. American Journal of Medical Genetics Part A. 2013;161(7):1755-8.

21. Henriques-Coelho T, Oliva-Teles N, Fonseca-Silva ML, Tibboel D, Guimarães H, Correia-Pinto J. Congenital diaphragmatic hernia in a patient with tetrasomy 9p. Journal of pediatric surgery. 2005;40(10):e29-e31.

22. Beck TF, Veenma D, Shchelochkov OA, Yu Z, Kim BJ, Zaveri HP, et al. Deficiency of FRAS1-related extracellular matrix 1 (FREM1) causes congenital diaphragmatic hernia in humans and mice. Human molecular genetics. 2012;22(5):1026-38.

23. Klaassens M, Scott D, van Dooren M, Hochstenbach R, Eussen H, Cai W, et al. Congenital diaphragmatic hernia associated with duplication of 11q23‐qter. American Journal of Medical Genetics Part A. 2006;140(14):1580-6.

24. Scott D, Cooper M, Stankiewicz P, Patel A, Potocki L, Cheung S. Congenital diaphragmatic hernia in WAGR syndrome. American Journal of Medical Genetics Part A. 2005;134(4):430-3.

25. Dworschak GC, Engels H, Becker J, Soellner L, Eggermann T, Kipfmueller F, et al. De Novo Duplication of 11p15 Associated With Congenital Diaphragmatic Hernia. Frontiers in Pediatrics. 2018;6.

26. Steiner MB, Vengoechea J, Collins RT. Duplication of the ALDH1A2 gene in association with pentalogy of Cantrell: a case report. Journal of medical case reports. 2013;7(1):287.

27. Van Esch H, Backx L, Pijkels E, Fryns J-P. Congenital diaphragmatic hernia is part of the new 15q24 microdeletion syndrome. European Journal of Medical Genetics. 2009;52(2-3):153-6.

28. Sharp AJ, Selzer RR, Veltman JA, Gimelli S, Gimelli G, Striano P, et al. Characterization of a recurrent 15q24 microdeletion syndrome. Human molecular genetics. 2007;16(5):567-72.

29. Wat MJ, Enciso VB, Wiszniewski W, Resnick T, Bader P, Roeder ER, et al. Recurrent microdeletions of 15q25. 2 are associated with increased risk of congenital diaphragmatic hernia, cognitive deficits and possibly Diamond–Blackfan anaemia. Journal of medical genetics. 2010;47(11):777-81.

30. Klaassens M, van Dooren M, Eussen H, Douben H, Den Dekker A, Lee C, et al. Congenital diaphragmatic hernia and chromosome 15q26: determination of a candidate region by use of fluorescent in situ hybridization and array-based comparative genomic hybridization. The American Journal of Human Genetics. 2005;76(5):877-82.

31. Biggio Jr JR, Descartes MD, Carroll AJ, Holt RL. Congenital diaphragmatic hernia: Is 15q26. 1‐26.2 a candidate locus? American Journal of Medical Genetics Part A. 2004;126(2):183-5.

32. Mosca A, Pinson L, Andrieux J, Copin H, Bigi N, Puechberty J, et al. Refining the critical region for congenital diaphragmatic hernia on chromosome 15q26 from the study of four fetuses. Prenatal diagnosis. 2011;31(9):912-4.

33. Genesio R, Maruotti GM, Saccone G, Mormile A, Conti A, Cicatiello R, et al. Prenatally diagnosed distal 16p11. 2 microdeletion with a novel association with congenital diaphragmatic hernia: A case report. Clinical case reports. 2018;6(4):592.

34. Hendrix NW, Clemens M, Canavan TP, Surti U, Rajkovic A. Prenatally diagnosed 17q12 microdeletion syndrome with a novel association with congenital diaphragmatic hernia. Fetal diagnosis and therapy. 2012;31(2):129-33.

35. Goumy C, Laffargue F, Eymard‐Pierre E, Kemeny S, Gay‐Bellile M, Gouas L, et al. Congenital diaphragmatic hernia may be associated with 17q12 microdeletion syndrome. American Journal of Medical Genetics Part A. 2015;167(1):250-3.

36. Sanford E, Bermudez‐Wagner K, Jeng L, Rauen KA, Slavotinek AM. Congenital diaphragmatic hernia in Smith–Magenis syndrome: A possible locus at chromosome 17p11. 2. American Journal of Medical Genetics Part A. 2011;155(11):2816-20.

37. Zayed H, Chao R, Moshrefi A, LopezJimenez N, Delaney A, Chen J, et al. A maternally inherited chromosome 18q22. 1 deletion in a male with late‐presenting diaphragmatic hernia and microphthalmia–evaluation of DSEL as a candidate gene for the diaphragmatic defect. American Journal of Medical Genetics Part A. 2010;152(4):916-23.

38. Petit F, Andrieux J, Holder-Espinasse M, Bouquillon S, Pennaforte T, Storme L, et al. Xq12q13. 1 microduplication encompassing the EFNB1 gene in a boy with congenital diaphragmatic hernia. European journal of medical genetics. 2011;54(5):e525-e7.

39. Chong K, Saleh M, Injeyan M, Miron I, Fong K, Shannon P. Nonisolated diaphragmatic hernia in Simpson‐Golabi‐Behmel syndrome. Prenatal diagnosis. 2018;38(2):117-22.

40. Scott TM, Campbell IM, Hernandez-Garcia A, Lalani SR, Liu P, Shaw CA, et al. Clinical exome sequencing data reveal high diagnostic yields for congenital diaphragmatic hernia plus (CDH+) and new phenotypic expansions involving CDH. Journal of Medical Genetics. 2021.

41. Kammoun M, Souche E, Brady P, Ding J, Cosemans N, Gratacos E, et al. Genetic profile of isolated congenital diaphragmatic hernia revealed by targeted next‐generation sequencing. Prenatal diagnosis. 2018;38(9):654-63.

42. Longoni M, High FA, Russell MK, Kashani A, Tracy AA, Coletti CM, et al. Molecular pathogenesis of congenital diaphragmatic hernia revealed by exome sequencing, developmental data, and bioinformatics. Proceedings of the National Academy of Sciences. 2014;111(34):12450-5.

43. Farrell SA, Sodhi S, Marshall CR, Guerin A, Slavotinek A, Paton T, et al. HLX is a candidate gene for a pattern of anomalies associated with congenital diaphragmatic hernia, short bowel, and asplenia. American Journal of Medical Genetics Part A. 2017;173(11):3070-4.

44. Slavotinek A, Moshrefi A, Lopez Jiminez N, Chao R, Mendell A, Shaw G, et al. Sequence variants in the HLX gene at chromosome 1q41‐1q42 in patients with diaphragmatic hernia. Clinical genetics. 2009;75(5):429-39.

45. Yu L, Sawle AD, Wynn J, Aspelund G, Stolar CJ, Arkovitz MS, et al. Increased burden of de novo predicted deleterious variants in complex congenital diaphragmatic hernia. Human molecular genetics. 2015;24(16):4764-73.

46. Ozdemir H, Plamondon J, Gaskin P, Asoglu MR, Turan S. A prenatally diagnosed case of Donnai‐Barrow syndrome: Highlighting the importance of whole exome sequencing in cases of consanguinity. American Journal of Medical Genetics Part A. 2019.

47. Beck TF, Campeau PM, Jhangiani SN, Gambin T, Li AH, Abo‐Zahrah R, et al. FBN1 contributing to familial congenital diaphragmatic hernia. American Journal of Medical Genetics Part A. 2015;167(4):831-6.

48. Balci TB, Strong A, Kalish JM, Zackai E, Maris JM, Reilly A, et al. Tatton‐Brown‐Rahman syndrome: Six individuals with novel features. American Journal of Medical Genetics Part A.

49. Birgmeier J, Esplin ED, Jagadeesh KA, Guturu H, Wenger AM, Chaib H, et al. Biallelic loss‐of‐function WNT5A mutations in an infant with severe and atypical manifestations of Robinow syndrome. American Journal of Medical Genetics Part A. 2018;176(4):1030-6.

50. Srour M, Chitayat D, Caron V, Chassaing N, Bitoun P, Patry L, et al. Recessive and dominant mutations in retinoic acid receptor beta in cases with microphthalmia and diaphragmatic hernia. The American Journal of Human Genetics. 2013;93(4):765-72.

51. Bleyl S, Moshrefi A, Shaw G, Saijoh Y, Schoenwolf G, Pennacchio L, et al. Candidate genes for congenital diaphragmatic hernia from animal models: sequencing of FOG2 and PDGFRα reveals rare variants in diaphragmatic hernia patients. European Journal of Human Genetics. 2007;15(9):950.

52. Jordan VK, Beck TF, Hernandez-Garcia A, Kundert PN, Kim B-J, Jhangiani SN, et al. The role of FREM2 and FRAS1 in the development of congenital diaphragmatic hernia. Human molecular genetics. 2018;27(12):2064-75.

53. Kaya TB, Aydemir O, Ceylaner S, Ceylaner G, Tekin AN. Isolated congenital diaphragm hernia associated with homozygous SLIT3 gene variant in dizygous twins. European Journal of Medical Genetics. 2021;64(7):104215.

54. Hosokawa S, Takahashi N, Kitajima H, Nakayama M, Kosaki K, Okamoto N. Brachmann‐de Lange syndrome with congenital diaphragmatic hernia and NIPBL gene mutation. Congenital anomalies. 2010;50(2):129-32.

55. Tuzovic L, Yu L, Zeng W, Li X, Lu H, Lu H-M, et al. A human de novo mutation in MYH10 phenocopies the loss of function mutation in mice. Rare diseases. 2013;1(1):e26144.

56. Piard J, Collet C, Arbez-Gindre F, Nirhy-Lanto A, Van Maldergem L. Coronal craniosynostosis and radial ray hypoplasia: a third report of Twist mutation in a 33 weeks fetus with diaphragmatic hernia. European journal of medical genetics. 2012;55(12):719-22.

57. Ackerman KG, Herron BJ, Vargas SO, Huang H, Tevosian SG, Kochilas L, et al. Fog2 is required for normal diaphragm and lung development in mice and humans. PLoS genetics. 2005;1(1):e10.

58. Arrington CB, Bleyl SB, Matsunami N, Bowles NE, Leppert TI, Demarest BL, et al. A family‐based paradigm to identify candidate chromosomal regions for isolated congenital diaphragmatic hernia. American Journal of Medical Genetics Part A. 2012;158(12):3137-47.

59. Yu L, Wynn J, Cheung YH, Shen Y, Mychaliska GB, Crombleholme TM, et al. Variants in GATA4 are a rare cause of familial and sporadic congenital diaphragmatic hernia. Human genetics. 2013;132(3):285-92.

60. Wat MJ, Beck TF, Hernández-García A, Yu Z, Veenma D, Garcia M, et al. Mouse model reveals the role of SOX7 in the development of congenital diaphragmatic hernia associated with recurrent deletions of 8p23. 1. Human molecular genetics. 2012;21(18):4115-25.

61. Longoni M, Lage K, Russell MK, Loscertales M, Abdul‐Rahman OA, Baynam G, et al. Congenital diaphragmatic hernia interval on chromosome 8p23. 1 characterized by genetics and protein interaction networks. American Journal of Medical Genetics Part A. 2012;158(12):3148-58.

62. Bulfamante G, Gana S, Avagliano L, Fabietti I, Gentilin B, Lalatta F. Congenital diaphragmatic hernia as prenatal presentation of Apert syndrome. Prenatal diagnosis. 2011;31(9):910.

63. Pinz H, Pyle LC, Li D, Izumi K, Skraban C, Tarpinian J, et al. De novo variants in Myelin regulatory factor (MYRF) as candidates of a new syndrome of cardiac and urogenital anomalies. American Journal of Medical Genetics Part A. 2018;176(4):969-72.

64. Hamanaka K, Takata A, Uchiyama Y, Miyatake S, Miyake N, Mitsuhashi S, et al. MYRF haploinsufficiency causes 46, XY and 46, XX disorders of sex development: bioinformatics consideration. Human molecular genetics. 2019.

65. Hucthagowder V, Sausgruber N, Kim KH, Angle B, Marmorstein LY, Urban Z. Fibulin-4: a novel gene for an autosomal recessive cutis laxa syndrome. The American Journal of Human Genetics. 2006;78(6):1075-80.

66. Antonius T, van Bon B, Eggink A, van der Burgt I, Noordam K, van Heijst A. Denys–Drash syndrome and congenital diaphragmatic hernia: Another case with the 1097G> A (Arg366His) mutation. American Journal of Medical Genetics Part A. 2008;146(4):496-9.

67. Schwab ME, Dong S, Lianoglou BR, Lucero AFA, Schwartz GB, Norton ME, et al. Exome sequencing of fetuses with congenital diaphragmatic hernia supports a causal role for NR2F2, PTPN11, and WT1 variants. The American Journal of Surgery. 2021.

68. Zarate Y, Zhan H, Jones J. Infrequent manifestations of Kabuki syndrome in a patient with novel MLL2 mutation. Molecular syndromology. 2012;3(4):180-4.

69. Reis LM, Tyler RC, Schilter KF, Abdul-Rahman O, Innis JW, Kozel BA, et al. BMP4 loss-of-function mutations in developmental eye disorders including SHORT syndrome. Human genetics. 2011;130(4):495-504.

70. Marguet F, Vezain M, Marcorelles P, Audebert-Bellanger S, Cassinari K, Drouot N, et al. Neuropathological hallmarks of fetal hydrocephalus linked to CCDC88C pathogenic variants. Acta neuropathologica communications. 2021;9(1):1-7.

71. Pasutto F, Sticht H, Hammersen G, Gillessen-Kaesbach G, FitzPatrick DR, Nürnberg G, et al. Mutations in STRA6 cause a broad spectrum of malformations including anophthalmia, congenital heart defects, diaphragmatic hernia, alveolar capillary dysplasia, lung hypoplasia, and mental retardation. The American Journal of Human Genetics. 2007;80(3):550-60.

72. Slavotinek AM, Moshrefi A, Davis R, Leeth E, Schaeffer GB, Burchard GE, et al. Array comparative genomic hybridization in patients with congenital diaphragmatic hernia: mapping of four CDH-critical regions and sequencing of candidate genes at 15q26. 1–15q26. 2. European journal of human genetics. 2006;14(9):999.

73. High FA, Bhayani P, Wilson JM, Bult CJ, Donahoe PK, Longoni M. De novo frameshift mutation in COUP‐TFII (NR2F2) in human congenital diaphragmatic hernia. American Journal of Medical Genetics Part A. 2016;170(9):2457-61.

74. Matsunami N, Shanmugam H, Baird L, Stevens J, Byrne JL, Barnhart DC, et al. Germline but not somatic de novo mutations are common in human congenital diaphragmatic hernia. Birth defects research. 2018;110(7):610-7.

75. Allen HL, Flanagan SE, Shaw-Smith C, De Franco E, Akerman I, Caswell R, et al. GATA6 haploinsufficiency causes pancreatic agenesis in humans. Nature genetics. 2012;44(1):20.

76. Yu L, Bennett JT, Wynn J, Carvill GL, Cheung YH, Shen Y, et al. Whole exome sequencing identifies de novo mutations in GATA6 associated with congenital diaphragmatic hernia. Journal of medical genetics. 2014;51(3):197-202.

77. Gaisl OC, Konrad D, Steindl K, Lang-Muritano M, editors. Novel Gata6-Mutation in a Boy with Neonatal Diabetes and Diaphragmatic Hernia. 57th Annual ESPE; 2018: European Society for Paediatric Endocrinology.

78. Brady P, Moerman P, De Catte L, Deprest J, Devriendt K, Vermeesch J. Exome sequencing identifies a recessive PIGN splice site mutation as a cause of syndromic congenital diaphragmatic hernia. European journal of medical genetics. 2014;57(9):487-93.

79. Alessandri J-L, Gordon CT, Jacquemont M-L, Gruchy N, Ajeawung NF, Benoist G, et al. Recessive loss of function PIGN alleles, including an intragenic deletion with founder effect in La Réunion Island, in patients with Fryns syndrome. European Journal of Human Genetics. 2018;26(3):340-9.

80. McInerney‐Leo AM, Harris JE, Gattas M, Peach EE, Sinnott S, Dudding‐Byth T, et al. Fryns syndrome associated with recessive mutations in PIGN in two separate families. Human mutation. 2016;37(7):695-702.

81. Yoo YS, Lee NH, Choi YB. Bochdalek hernia with Diamond-Blackfan anemia associated with RPS19 gene mutation: A case report. Medicine. 2019;98(39):e17337.

82. Urban Z, Hucthagowder V, Schürmann N, Todorovic V, Zilberberg L, Choi J, et al. Mutations in LTBP4 cause a syndrome of impaired pulmonary, gastrointestinal, genitourinary, musculoskeletal, and dermal development. The American Journal of Human Genetics. 2009;85(5):593-605.

83. Thomas E, Lewis AM, Yang Y, Chanprasert S, Potocki L, Scott DA. Novel Missense Variants in ADAT3 as a Cause of Syndromic Intellectual Disability. Journal of pediatric genetics. 2019;8(04):244-51.

84. Hogue J, Shankar S, Perry H, Patel R, Vargervik K, Slavotinek A. A novel EFNB1 mutation (c. 712delG) in a family with craniofrontonasal syndrome and diaphragmatic hernia. American Journal of Medical Genetics Part A. 2010;152(10):2574-7.

85. Vasudevan PC, Twigg SR, Mulliken JB, Cook JA, Quarrell OW, Wilkie AO. Expanding the phenotype of craniofrontonasal syndrome: two unrelated boys with EFNB1 mutations and congenital diaphragmatic hernia. European journal of human genetics. 2006;14(7):884.

86. Dias C, Basto J, Pinho O, Barbêdo C, Mártins M, Bornholdt D, et al. A nonsense porcn mutation in severe focal dermal hypoplasia with natal teeth. Fetal and pediatric pathology. 2010;29(5):305-13.

87. Verma S, Purrier S, Breidbart E, Pappas JG, Mally PV, Randis TM. Hyponatremic Seizures and Adrenal Hypoplasia Congenita in a Neonate with Congenital Diaphragmatic Hernia. Case Reports in Pediatrics. 2019;2019.

88. Kelly M, von Lintig J. STRA6: role in cellular retinol uptake and efflux. Hepatobiliary surgery and nutrition. 2015;4(4):229.

89. Hind M, Corcoran J, Maden M. Alveolar proliferation, retinoid synthesizing enzymes, and endogenous retinoids in the postnatal mouse lung: different roles for Aldh-1 and Raldh-2. American journal of respiratory cell and molecular biology. 2002;26(1):67-73.

90. Volpe MV, Wang KTW, Nielsen HC, Chinoy MR. Unique spatial and cellular expression patterns of Hoxa5, Hoxb4, and Hoxb6 proteins in normal developing murine lung are modified in pulmonary hypoplasia. Birth Defects Research Part A: Clinical and Molecular Teratology. 2008;82(8):571-84.

91. Bartholin L, Powers SE, Melhuish TA, Lasse S, Weinstein M, Wotton D. TGIF inhibits retinoid signaling. Molecular and cellular biology. 2006;26(3):990-1001.

92. Cooney AJ, Tsai SY, O'Malley BW, Tsai M. Chicken ovalbumin upstream promoter transcription factor (COUP-TF) dimers bind to different GGTCA response elements, allowing COUP-TF to repress hormonal induction of the vitamin D3, thyroid hormone, and retinoic acid receptors. Molecular and cellular biology. 1992;12(9):4153-63.

93. Qiu Y, Krishnan V, Pereira F, Tsai S, Tsai M-J. Chicken ovalbumin upstream promoter-transcription factors and their regulation. The Journal of steroid biochemistry and molecular biology. 1996;56(1-6):81-5.

94. Huggins GS, Bacani CJ, Boltax J, Aikawa R, Leiden JM. Friend of GATA 2 physically interacts with chicken ovalbumin upstream promoter-TF2 (COUP-TF2) and COUP-TF3 and represses COUP-TF2-dependent activation of the atrial natriuretic factor promoter. Journal of Biological Chemistry. 2001;276(30):28029-36.

95. Clugston RD, Zhang W, Greer JJ. Gene expression in the developing diaphragm: significance for congenital diaphragmatic hernia. American Journal of Physiology-Lung Cellular and Molecular Physiology. 2008;294(4):L665-L75.

96. Iritani I. Experimental study on embryogenesis of congenital diaphragmatic hernia. Anatomy and embryology. 1984;169(2):133-9.

97. Sefton EM, Gallardo M, Kardon G. Developmental origin and morphogenesis of the diaphragm, an essential mammalian muscle. Developmental biology. 2018;440(2):64-73.

98. Dietrich S, Abou-Rebyeh F, Brohmann H, Bladt F, Sonnenberg-Riethmacher E, Yamaai T, et al. The role of SF/HGF and c-Met in the development of skeletal muscle. Development. 1999;126(8):1621-9.

99. Bladt F, Riethmacher D, Isenmann S, Aguzzi A, Birchmeier C. Essential role for the c-met receptor in the migration of myogenic precursor cells into the limb bud. Nature. 1995;376(6543):768.

100. Kosiński P, Wielgoś M. Congenital diaphragmatic hernia: pathogenesis, prenatal diagnosis and management—literature review. Ginekologia polska. 2017;88(1):24-30.

101. Dott MM, Wong LYC, Rasmussen SA. Population‐based study of congenital diaphragmatic hernia: risk factors and survival in Metropolitan Atlanta, 1968–1999. Birth Defects Research Part A: Clinical and Molecular Teratology. 2003;67(4):261-7.

102. Tonks A, Wyldes M, Somerset D, Dent K, Abhyankar A, Bagchi I, et al. Congenital malformations of the diaphragm: findings of the West Midlands Congenital Anomaly Register 1995 to 2000. Prenatal Diagnosis: Published in Affiliation With the International Society for Prenatal Diagnosis. 2004;24(8):596-604.
